# Large-scale Vietnamese point-of-interest classification using weak labeling

**DOI:** 10.3389/frai.2022.1020532

**Published:** 2022-12-09

**Authors:** Van Trung Tran, Quang Dao Le, Bao Son Pham, Viet Hung Luu, Quang Hung Bui

**Affiliations:** ^1^Center of Multidisciplinary Integrated Technologies for Field Monitoring, Vietnam National University of Engineering and Technology, Hanoi, Vietnam; ^2^NTT Hi-Tech Institute, Nguyen Tat Thanh University, Ho Chi Minh City, Vietnam; ^3^Faculty of Information Technology, VNU University of Engineering and Technology, Hanoi, Vietnam; ^4^FIMO, Hanoi, Vietnam

**Keywords:** crowd-sourcing, point-of-interest, weak labeling, snorkel, BERT-based

## Abstract

Point-of-Interests (POIs) represent geographic location by different categories (e.g., touristic places, amenities, or shops) and play a prominent role in several location-based applications. However, the majority of POIs category labels are crowd-sourced by the community, thus often of low quality. In this paper, we introduce the first annotated dataset for the POIs categorical classification task in Vietnamese. A total of 750,000 POIs are collected from WeMap, a Vietnamese digital map. Large-scale hand-labeling is inherently time-consuming and labor-intensive, thus we have proposed a new approach using weak labeling. As a result, our dataset covers 15 categories with 275,000 weak-labeled POIs for training, and 30,000 gold-standard POIs for testing, making it the largest compared to the existing Vietnamese POIs dataset. We empirically conduct POI categorical classification experiments using a strong baseline (BERT-based fine-tuning) on our dataset and find that our approach shows high efficiency and is applicable on a large scale. The proposed baseline gives an F1 score of 90% on the test dataset, and significantly improves the accuracy of WeMap POI data by a margin of 37% (from 56 to 93%).

## 1. Introduction

With the increasing availability of consumer-graded geo-location devices (e.g., GPS-equipped smartphones), and the development of location-sharing platforms (e.g., Google Places and Foursquare), Volunteered Geographic Information (VGI) has been emergently generated by large numbers of private citizens (Goodchild, 2007). Despite that being used in an increasing number of online mapping applications (e.g., Google Maps and OpenStreetMap), VGI suffers from quality and reliability issues due to uncontrolled contributor's levels of expertise (Basiri et al., 2019).

As a major part of VGI projects, Points-of-Interests (POIs) describe geographic locations, such as tourist places, amenities, or shops, and are largely crowd-sourced by citizens (Touya et al., 2017). Due to its key role in many popular consumer applications (e.g., Facebook and Swarm), assessing the quality of VGI POI is crucial and is one of the main research topics related to VGI. A common practice for POI quality assessment is to automatically (re-)classify their categorical classes and evaluate the accuracy of crowd-sourced labels. POI classification can be defined as a straightforward multi-class classification problem (Giannopoulos et al., 2019). Several algorithms have been proposed to classify POI and can be roughly divided into two main categories: semi-automatic methods that assist the user during the manual annotation process and supervised machine learning/deep learning (ML/DL) based approaches.

Context-based approaches are often used to assist the user during the annotation process. These approaches rely on Tobler's first law of geography stating that “all things are related, but nearby things are more related than distant things” (Tobler, 1970; Vandecasteele and Devillers, 2015). A methodology to analyze the spatial-semantic interaction of point features was proposed by Mülligann et al. (2011). By analyzing the spatial co-occurrence, potential correlations between two POIs of different types or the same type are identified. Their results set the stage for systems that assist VGI contributors in suggesting the types of new point features (Mülligann et al., 2011). Another user-assisted tag recommendation system for POI categories was proposed by Vandecasteele and Devillers (2015) as a plugin for the Java OpenStreetMap editor (JOSM). The plugin exploits the co-occurrence of historical POI categories and suggests a list of related categories depending on the categories already specified for the selected POIs.

Most of the ML-based POI classification works are based on two steps. First, a meaningful representation of POI using its metadata (e.g., name, address, and coordinates) are extracted as training features. Then, popular conventional machine learning algorithms, such as SVM (Crammer and Singer, 2002) and k-Nearest Neighbor (Goldberger et al., 2004), are deployed to learn classification models. An approach for automatically recommending categories with a set of textual, spatial, and semantic features was proposed by Giannopoulos et al. (2016). Four different classification ensembles, namely SVM, kNN, clustering+SVM and clustering+kNN, were tested and achieved 60% accuracy for top-1 recommendation on 1400 categories. A POI categorization method that incorporates both onomastic and local contextual information as POI features was proposed by Choi et al. (2014). Although the method achieved high accuracy of 73.053%, their method requires additional POI contextual information, such as online reviews, thus not applicable for a large-scale setting where an abundance of metadata is not usually available. Recent studies proposed a new approach where minimum metadata of POI including names and coordinates are used (Giannopoulos et al., 2019; Eftaxias et al., 2019). A set of textual and neighborhood-based features directly derived from names and coordinates of POI are proposed. Textual features are defined by the bag-of-term and n-grams (including 1-grams, 3-grams, and 4-grams) frequency calculated from the POI name. Meanwhile, neighborhood-based features measure the co-occurrence of different POI categories in a nearby neighborhood. A series of eight ML algorithms including Naive Bayes, k-NN, SVM, Logistic Regression, MLP, Decision Tree, Random Forest, and Extra Trees are tested where Extra Trees achieved the highest accuracy of 78.8% on Level-1 categories (13 classes) and 67.9% on Level-2 categories (32 classes), respectively. These methods, however, still heavily rely on additional resources of nearby true-label POI categories for neighborhood-based features. With the recent advances in DL for natural language processing, especially pre-trained language models, the automatic classification of POI based only on textual features from POI is becoming feasible. Zhou et al. (2020) proposed a convolutions neural network (CNN) model to solve the POI classification problem using POI's name and external text knowledge searched from the internet. Word2Vec language model (Mikolov et al., 2013) was utilized to obtain word vectors with dimensions of 300 for each POI's name and external text knowledge; then these two-word embeddings were passed on to an attention-based head network to obtain the prediction result.

While ML/DL works well for tasks with sufficient data, gathering enough training labels is a major impediment in applying ML/DL to real-world applications (Ratner et al., 2017; Varma and Ré, 2018). Giannopoulos et al. (2019) conducted their experiments using a proprietary POI dataset on a Greek city (Marousi) which contain only 884 POIs in 13 first-level categories. Zhou et al. (2020) selected the first-level POI dataset for Beijing which included 16 categories and 133,116 samples^1^. Among others, Yelp Open Dataset^2^ is considered the most popular dataset for POI classification which contains 150,346 POIs in 11 metropolitan areas.

Notably, existing datasets for the POI classification task are all in English. To the best of our knowledge, there is little to no POI dataset available for low-resource languages such as Vietnamese. As a result, our work aims to achieve the following main goals: **(i)** to provide a new dataset for the categorization of POI in low-resource languages, particularly Vietnamese and **(ii)** to provide an effective framework for POI classification using weak-labeling to reduce the cost of the data annotation process. Overall, our contributions are summarized as follows:

- We introduce the first large-scale Vietnamese POI dataset for the multi-label classification task. Our dataset is annotated with 15 different classes related to POI types in Vietnam, consisting of 720,000 crowd-sourced noisy-labeled POIs for training and 30,000 gold-standard annotated POIs for testing.
- We explore the weak-labeling process and show that using simple heuristic rules is effective for generating large-scale training corpora and learning a powerful classification network.
- We publicly release our dataset for research or educational purposes. We hope that our dataset can serve as a starting point that potentially impacts research and real-world applications where POI plays an important role.

## 2. Materials and methods

### 2.1. Data collection

**Figure 1:**
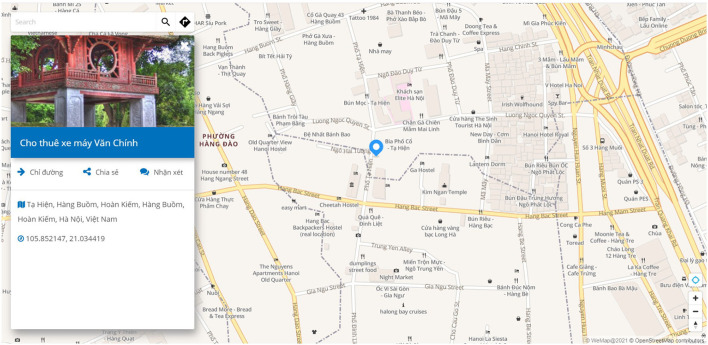
Example of Point-of-Interests (POIs) distribution on WeMap. Each POI contains a name, address, and geographic location.

In this study, POI data is partially derived from WeMap, a Vietnamese map platform. WeMap currently contains 25.3 million Vietnamese POIs that are crowd-sourced by the community using smartphone-based geo-survey application. POI data contain name, address, coordinates, POI categories, administrative boundaries of POI, and other metadata such as popularity, phone number, and opening-hour. Figure 1 shows an example of POIs information on WeMap with name, address, and geographic location.

**Figure 2:**
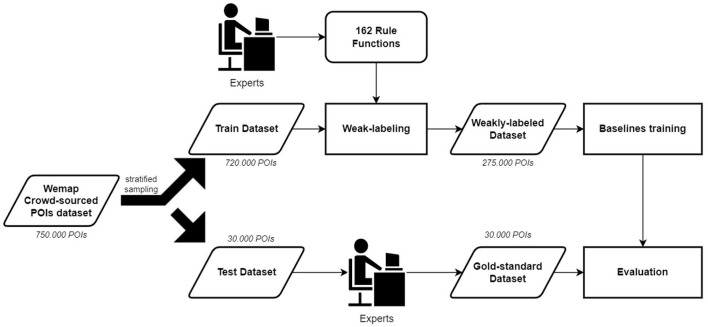
The proposed approach overview.

Originally, POIs from WeMap are categorized into 162 classes using a custom taxonomy defined by VNPost^3^, a largest postal service company in Vietnam. We then mapped these categories into 15 categories based on the Pelias first-level taxonomy^4^ including accommodation, education, entertainment, finance, food, government, health, industry, natural, nightlife, professional, recreation, religion, retail, and transport. A total of 750,000 POIs are retrieved from the WeMap database. Only the name and original crowd-sourced categories of POI are kept, while other metadata are removed. Stratified sampling on crowd-sourced categories is used to split the dataset into two parts. The first part contains 30,000 POIs that are used to create gold-standard testing sets. Meanwhile, the rest of the 720,000 POIs are used to create a weak-labeled training set as described in Section 2.2. Details of the data flow are shown in Figure 2.

### 2.2. Weak-labeling

Snorkel, a programmatic data labeling tool, is initialized for a weak-labeling process (Ratner et al., 2017; Varma and Ré, 2018; Bach et al., 2019). The labeling functions are used to assign labels to all samples that satisfy its heuristics patterns, rather than manually labeling every sample one by one. Such an approach allows us to express various weak supervision sources of labeling functions including the following:

- **Keyword matching** looks for a specific keyword in the text.
- **Pattern matching** looks for a specific syntactical pattern.
- **Distant supervision** uses an external knowledge base.
- **Model-based** labeling functions use the predictions of pre-trained models (usually a model for a different task other than the one at hand)
- **Crowdworker labels** treats each crowd worker as a black-box function that assigns labels to subsets of the data.

An initial template of the labeling function is developed using the keyword matching approach. In its most general form, a labeling function is a simple function written in Python which accepts a candidate PoI's name and a list of heuristic keywords as input. The appearance of keywords in the PoI's name indicates a label or abstains. The template code for keyword-matching labeling functions is illustrated below:


from snorkel.labeling import labeling_function                         @labeling_function()                  def keywords_matching_function(x, keywords):    return 1 if any(word **in** x.text.lower() for     word in keywords) else -1


**Figure 3:**
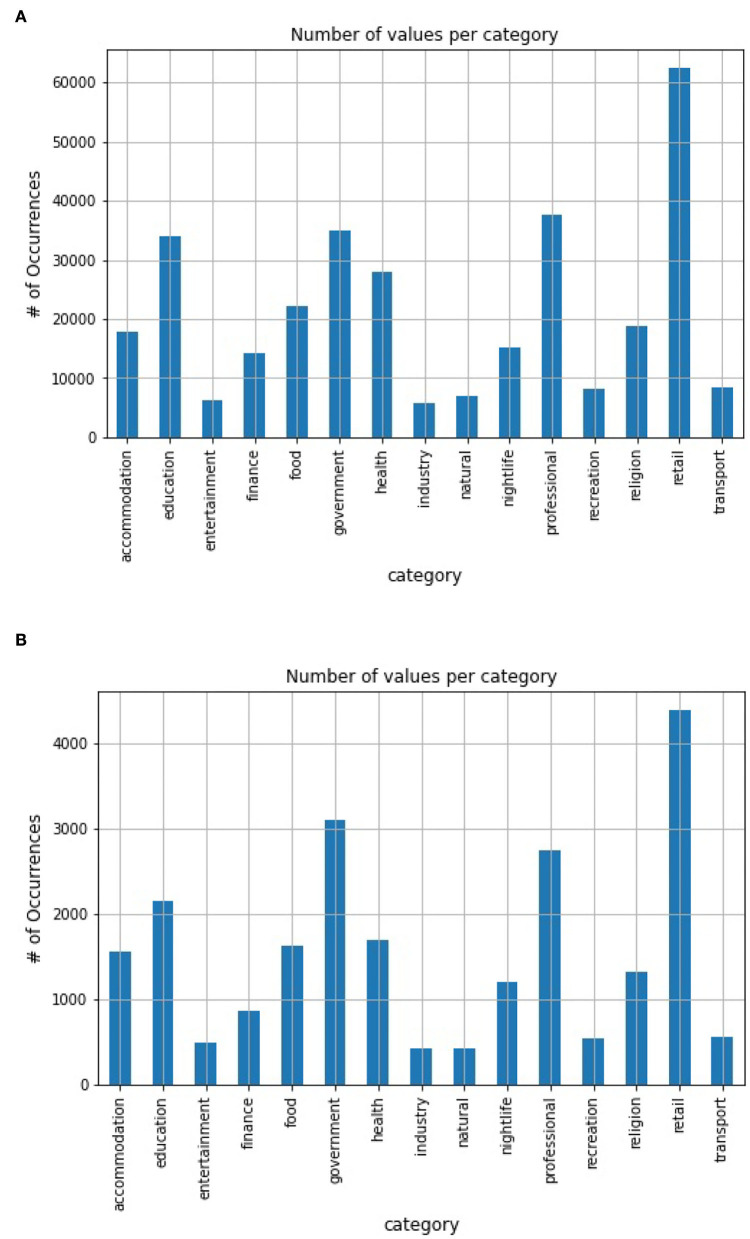
Dataset statistics. **(A)** Weakly-labeled training set distribution. **(B)** Gold-standard testing set distribution.

A total of 6 annotators with strong linguistic abilities are employed. Each annotator could write 27 labeling functions, thus resulting in 162 labeling functions. A generative model, which is essentially a re-weighted combination of the provided labeling functions, is then learned. It requires no ground truth and thus can be learned instead from the agreements and disagreements of the labeling functions (Varma and Ré, 2018). Through this process, a set of 275,000 POIs with high-confidence probabilistic labels is derived and used as training data for the later discriminative baseline model. Statistics of our training dataset are presented in Figure 3A. During the training, 10% of data were selected as a validation set using stratified sampling.

### 2.3. Baselines training

Our ultimate goal is to train a model that generalizes beyond the information expressed in the above weakly labeling functions. We conduct experiments on our generated probabilistic labels dataset using a strong discriminative model to investigate (i) the performance of large-scale weak-labeled data, and (ii) the effectiveness of large pre-trained language models on the short-text classification task. The baselines include PhoBERT (Nguyen and Tuan Nguyen, 2020) and viBERT (Bui et al., 2020) which are monolingual variants on the Vietnamese datasets of BERT and RoBERTa, respectively. PhoBERT is pre-trained on a 20GB text corpus combining Vietnamese Wikipedia and Vietnamese news, while viBERT is pre-trained on 10GB of news corpus.

Several pre-processing steps are used including character case conversion and word segmentation. Since the name of POI often contains proper names, which does not improve prediction efficiency, we decided to perform a conversion of all POI names to lowercase. RDRSegmenter (Vu et al., 2018) is applied to perform automatic Vietnamese word segmentation.

Both PhoBERT and viBERT are fine-tuned using the *transformers* library (Wolf et al., 2020). AdamW optimizer with a fixed learning rate of 2e-05 and a batch size of 32 is used. The baselines are fine-tuned for 30 epochs with early stopping if no performance improvement is found after 5 continuous epochs. Micro-average F1 score on the validation set is used to select the best model checkpoint to report the final score. Details of the hyper-parameter that we used for both PhoBERT and viBERT are presented in Table 1.

**Table 1:** Hyper-parameters.

**Hyperparameter**	**Value**
Dropout	0.5
Mixout	True
Freeze Encoder	False
Epochs	5
Scheduler	constant_schedule_with_warmup
Restored Epoch	1
TrainBatch Size	32
Optimizer	AdamW
Learning rate	2e05
Weight decay	0.01
Monitor	val_loss
Mode	min
Patience	3
Min delta	–0.001

## 3. Results

We conducted experiments using baselines on our gold-standard test set of 30,000 POIs. Following Brandsen et al. (2020), a gold-standard test set was created by a two-phase annotation process. First, two guideline annotators randomly annotated 10% of the test set independently. Then, five annotators were employed to annotate the whole test set. Annotations are revised until they achieve an inter-annotator agreement *F*1 score of at least 0.92 calculated over the POIs that already have gold annotations from the 10% set. Statistics of our test set can be found in Figure 3B.

Table 2 shows the classification results of the baselines on the test set. In addition to the standard micro-average F1 score, we also reported the macro-average F1 score. We found that both PhoBERT and viBERT produced desirable performances thus confirming the effectiveness of large-scale weak-labeled training using pre-trained monolingual language models on the short-text classification task. Overall, PhoBERT outperformed viBERT (Weighted-F1: 0.90 vs. 0.87; Macro-F1: 0.88 vs. 0.86). We performed an error analysis using the best-performing model, PhoBERT, and found the following observations:

- There are some categories with low *F*_1_ score (i.e., 63% for *recreation*). It is largely due to the fact that these categories have a significantly lower number of data points compared to others, thus resulting in unbalance problem.
- The appearance of abbreviation words, specifically uncommon ones, significantly reduces the performance of baselines, e.g., “*trung tâm vhtt xã hiẽu liêm”* (in English “*Hieu Liem commune cultural and sports center,”* here “*trung tâm vhtt”* is an abbreviation for “*trung tâm v*ă*n hóa thẽ thao,”* which means “*cultural and sports center”*). “*trung tâm v*ă*n hóa thẽ thao”* is the key semantic content for POI type reasoning; however, its abbreviation “*trung tâm vhtt”* provides no meaning.
- The baselines is struggling to differentiate ambiguous categories such as *professional* vs. *retail* and *food* vs. *retail*. For example, “*quang linh mobile”* (Quang Linh mobile store) and “*nam long mobile”* (Nam Long mobile store) should be of category *retail* but are predicted as *professional*.

**Table 2:** Baselines *F*_1_ score for each type of Point-of-Interests (POIs), macro-average, and weighted-average *F*_1_ scores.

	**PhoBERT**			**viBERT**		
	**Precision**	**Recall**	**F1**	**Precision**	**Recall**	**F1**
Accommodation	0.92	0.92	0.92	0.97	0.94	**0.95**
Education	0.97	0.93	**0.95**	0.96	0.94	**0.95**
Entertainment	0.76	0.90	**0.82**	0.80	0.80	0.80
Finance	0.98	0.95	0.96	0.99	0.97	**0.98**
Food	0.99	0.67	**0.80**	0.97	0.36	0.53
Government	0.97	0.92	0.95	0.97	0.94	**0.96**
Health	0.96	0.98	0.97	0.98	0.99	**0.98**
Industry	0.78	0.92	0.85	0.85	0.90	**0.88**
Natural	0.96	0.91	**0.93**	0.87	0.92	0.89
Nightlife	0.68	0.97	0.80	0.93	0.99	**0.96**
Professional	0.93	0.82	0.87	0.94	0.82	**0.88**
Recreation	0.53	0.76	**0.63**	0.39	0.73	0.50
Religion	0.98	0.93	**0.96**	0.97	0.96	**0.96**
Retail	0.72	0.96	**0.82**	0.61	0.96	0.75
Transport	0.98	0.89	0.93	0.96	0.94	**0.95**
**Macro avg**	0.87	0.90	**0.88**	0.88	0.88	0.86
**Weighted avg**	0.91	0.90	**0.90**	0.91	0.88	0.87

Table 3 evaluates the improvement gained from our generated POI categories using the best performing model PhoBERT compared with the original crowd-sourcing categories. Our proposed approach has significantly improved the accuracy of POI categories from 56 to 93%.

**Table 3:** Accuracy evaluation of POI based on original WeMap's crowd-sourcing categories compared to automatically generated categories from our proposed approach.

	**Crowd-sourcing**	**Proposed approach (PhoBERT)**
Accommodation	0.37	**0.94**
Education	0.69	**0.97**
Entertainment	0.58	**0.88**
Finance	0.71	**0.96**
Food	0.53	**0.90**
Government	0.67	**0.93**
Health	0.58	**0.98**
Industry	0.25	**0.89**
Natural	0.69	**0.95**
Nightlife	0.53	**0.89**
Professional	0.46	**0.91**
Recreation	0.67	**0.84**
Religion	0.63	**0.95**
Retail	0.39	**0.96**
Transport	0.67	**0.93**
**Average accuracy**	0.56	**0.93**

## 4. Discussion

In this paper, we presented the first results from deploying the weak labeling process in a large-scale, industrial setting for the POI classification problem. We empirically conducted experiments on our dataset and showed that weak labeling significantly increases the efficiency of the pre-trained language models and reduces the time and effort of hand-labeling for a large amount of training data. We hope that our findings can be a starting point for NLP research and applications in the POI domain in the future.

## Data availability statement

The datasets presented in this study can be found in online repositories. The names of the repository/repositories and accession number(s) can be found below: https://github.com/PIVASIA/wemap-poi-dataset.

## Author contributions

LH, PS, and BH contributed to the conception and design of the study. TT conducted the experiments. LD organized the dataset. LH wrote the first draft of the manuscript. TT, LD, BH, and PS wrote sections of the manuscript. All authors contributed to the manuscript revision, read, and approved the submitted version.

## Footnotes

^1^ http://www.poi58.com/search/s/download/beijingpois.html

^2^ https://www.yelp.com/dataset

^3^ http://www.vnpost.vn/

^4^ https://github.com/pelias/pelias/wiki/Taxonomy-v1#pelias-taxonomy-v1-proposal
